# Supplementary material for: Patterns of Intron Gain and Loss in Fungi
Source: PLoS Biol. 2004 Nov 30;2(12):e422. doi: 10.1371/journal.pbio.0020422 (PMC532390; doi:10.1371/journal.pbio.0020422)
Supplement: Table S1 — Also available at http://genes.mit.edu/NielsenEtAl/. (4.3 MB ZIP). [file pbio.0020422.st001.zip › NielsenEtAl/html/1276.html]

AN7313.1.NCU06861.1.MG03844.1.FG11366.1


```
 CLUSTAL W (1.82) Multiple Sequence Alignments - Introns Inserted


Sequence 1: AN7313.1	475 aa
Sequence 2: FG11366.1	447 aa
Sequence 3: NCU06861.1	363 aa
Sequence 4: MG03844.1	470 aa
Alignment Length: 505 aa
Number Identitical Residues: 135 aa
Alignment Score (without introns) 8195


MG03844.1 	------MLALRRSATLGLAILSGAYASLQIVPGGTWTA0DN-GQHIQAHGTGI-------
NCU06861.1	MHHRNRLLLSLLSVIMGLATST-ALAALQIVPGGTWTA0SN-GEHLNAHGAGV-------
FG11366.1 	-------MLHYLSALVAALAASTVQASADIVSGATWTA0TNTGEHVQAHGHGL-------
AN7313.1  	-------MHRRFKILYFALVAKYARAALDIIPGATWTA0DSTSKHMAPDSCMISCLPSPQ
          	       :    .        . . *: :*:.*.****  .:.:*: ...  ::. .:..

MG03844.1 	-------T~VVNGTYFMIGEDKTNGSAFQNVNCYSSTDLVRWHYEGSLLSRTAEAG-DLG
NCU06861.1	-------I~QVNNTFYLIGEDKSQGSSFFAINCYSSVDLVQWTYEGALLSRTTSSG-DLG
FG11366.1 	-------I2EVDGTYYMIGEDKTDGTFFQNVNCYSSTDLVEWTYRGALLSRTSEAG-DLG
AN7313.1  	YQPLTLHS~EEDGTYYMIGEEKTDGALFQAVNCYSSTNLVEWTFVNQLLTVTEEEGADLG
          	 .. :      :.*:::***:*::*: *  :*****.:**.* : . **: * . *:***

MG03844.1 	PNRIVERPKVIFNERTQKYVLWMHIDSSDYKDARAGVAVGDSVCGK-FT1YLGSSRPLGF
NCU06861.1	PNRIIERPKVIYNDLTKKYVMWMHVDSSNYGDAKVGVATSDTVCGKPYK~YHGSFRPLGM
FG11366.1 	PNRIVERPKVIYNDDTKKYVLYLHIDSSDYKDARVGVATGDSVCGK-YT~YHRSFRPLGK
AN7313.1  	PNRIVERPKVIKNDNTGQYVMWMHIDSTDYADARVGIATGNSVCED-YV~YRESFRPLGF
          	****:****** *: * :**:::*:**::* **:.*:*..::** . :  *  * **** 

MG03844.1 	QSRDSGLFKDDD--GTAYLLTED0LTHRKKPPSTAQREHGLRINRLTDDYLGFADNTSTY
NCU06861.1	ESRDIGLFKDDDDDGRAYLLTED0------------RKNGLRINPLTPDYLSISGTTSTH
FG11366.1 	QSRDMGLFKDDD--GSAYLMTED0------------REYGTRIMALSDDYLNVTEIT--Y
AN7313.1  	QSRDIGLFKDED--GSAYLLSED0------------REYGTRIIRLTDDYLHVANVT--F
          	:*** *****:*  * ***::**             *: * **  *: *** .:  *  .

MG03844.1 	LWSESIESPAMLKRNGTYYMFGSKLTGWDPNDN~VYSTATSISGPWSGWQTFADKGSNTY
NCU06861.1	LFPTAIESPALLKLSGRYFLFGSHLTGWSPNDN~VYTTSTNLSGPWTDWKVFADKGSNTY
FG11366.1 	EWKYFAESPAMLKQNGYYFIFGSHLTGWDANDN0VYSYAKSLSGPWSAWTEFAPVGSKTY
AN7313.1  	GWEYFAESPALIKRGETYFIFGSHLTGWSPNDN0IYSTATSLSGPWTDWTEVAPVGSNTF
          	 :    ****::* .  *::***:****..*** :*: :..:****: *  .*  **:*:

MG03844.1 	ASQTTYILPYGDGGNVMYLGDRWVSTNLQSSTYIWLPLDISGTTVS----LKNRDAWIPN
NCU06861.1	ASQTSFILNYGGPGNIMYMGDRWRSSNLQSSSYIWLPLNISDTTTTTVVLLKNRASWIPN
FG11366.1 	QSQVSYIQPLGN-GNAIYIGDRWVSTNLAASTYVWLPLKVEGTKVT----LSWYDSWSPN
AN7313.1  	SSQVNYVLPLGT-DKAIYMGDRWVSSNLGASTYIWLPLQIDGTTATAS--LTWYDSWSVD
          	 **..::   *  .: :*:**** *:** :*:*:****.:..*..::   *.   :*  :

MG03844.1 	AAPPGAPWQPAGDVNAYEGEVAQLGGGARV~VSCSGCSAGSAAGYIGGDVNG-----IVT
NCU06861.1	AQS-GLLWADSPAEKSYEGERGSYAGGAKE0HHHLKHTK---------------------
FG11366.1 	VSK--GTW-SETKMTKLEGENAVMANDARV~ISCNECSGSKAVGYIGGDKKG--------
AN7313.1  	LAS--GTWNAEEDETVYEGEDGALSGGARL~IDCSRCSGSTAAGYIGGAGDGENDGAVTL
          	       *      .  *** .  ...*:        : ..: .  ..  ......:   

MG03844.1 	ISGVRSSAPDGTLTTVRIRFANGDGNPRYANVTVNGGPPTRLAFEPTRGGVSDSTLNVQL
NCU06861.1	------------------------------------------------------------
FG11366.1 	TLTFKSVKSSGGLSTINIKYRNGDTGSRYATVSVNG-KSQKLAFLSTRHLSQTGLSRGFF
AN7313.1  	QVDYATQSTDSARITLNIHYLNGDTDPRYAAVSVNGGEAQTIAFLSTNQLSGSGSSAVHV
          	     :  ...   :      ... ..  :  : ... .   :  .:      .      

MG03844.1 	RP-STDNTIVVSGAGGGLWGPDVDRLIVPVD
NCU06861.1	-------------------------------
FG11366.1 	DLEEGDNTITIS--NNDGWSPDVDALLPPVA
AN7313.1  	DLVQGVNTVQISGVEGGGWGPDIDQVAVPL-
          	   .  .:   :.  ..  ... .    .
```
